# Are affective temperaments associated with seasonal pattern in mood disorders? Results from a cross-sectional study

**DOI:** 10.1192/j.eurpsy.2026.10708

**Published:** 2026-07-31

**Authors:** M. Di Vincenzo, B. Della Rocca, S. Cipolla, C. Toni, S. Calamaro, F. Guida, G. Sampogna, M. Luciano, A. Fiorillo

**Affiliations:** Department of Psychiatry, University of Campania “Luigi Vanvitelli”, Naples, Italy

## Abstract

**Introduction:**

Major depressive disorder (MDD) and bipolar disorder (BD) are challenging and disabling conditions, whose clinical management requires thorough assessment and characterization of each patient’s psychopathological status. Several clinical correlates, including affective temperaments and seasonal pattern, have been found in association with the severity levels of both disorders. However, their association has not yet been examined in depth.

**Objectives:**

This study aims to explore how affective temperaments influence the seasonal pattern of illness in patients suffering from MDD or BD.

**Methods:**

Patients diagnosed with MDD and BD types I and II according to the DSM-5 criteria were consecutively enrolled at the Department of Psychiatry of University of Campania “Luigi Vanvitelli” in Naples, Italy. Affective temperaments were assessed using the brief version of the Munster Temperament Evaluation of the Memphis, Pisa, Paris and San Diego (b-TEMPS-M) scale; seasonality was investigated using the Seasonal Pattern Assessment Questionnaire (SPAQ). Anxiety levels, depressive and manic symptoms, trait impulsivity, quality of life, and personal and social functioning were assessed as secondary outcomes using standardized tools. Univariate and linear regression analyses were carried out in order to test the association between the variables.

**Results:**

The study included 100 patients, mainly women (68%), with a mean age of 45.1±14.0 years. Among them, 66 were diagnosed with MDD and 34 suffered from BD. A seasonal pattern — defined by a Global Seasonality Score (GSS) greater than 10 on the SPAQ — was observed in 47 patients. Higher GSS values significantly correlated with depressive (p<.01), anxious (p<.01), and cyclothymic (p< .01) temperament scores on the b-TEMPS-M. Linear regression analysis further confirmed associations between GSS and depressive (p < .01) and anxious (p < .01) temperaments, as well as trait impulsivity (p < .05).

**Conclusions:**

Depressive and anxious temperaments are associated with higher risk of suffering from MDD or BD with seasonal pattern. The evaluation of affective dispositions and seasonality could optimize the clinical characterization of patients with mood disorders, in order to provide targeted and tailored treatments.

**Disclosure of Interest:**

None Declared

